# Predictable internal brain dynamics in EEG and its relation to conscious states

**DOI:** 10.3389/fnbot.2014.00018

**Published:** 2014-06-03

**Authors:** Jaewook Yoo, Jaerock Kwon, Yoonsuck Choe

**Affiliations:** ^1^Department of Computer Science and Engineering, Texas A&M UniversityCollege Station, TX, USA; ^2^Department of Electrical and Computer Engineering, Kettering UniversityFlint, MI, USA

**Keywords:** predictable dynamics, consciousness, neuroevolution, EEG, sleep

## Abstract

Consciousness is a complex and multi-faceted phenomenon defying scientific explanation. Part of the reason why this is the case is due to its subjective nature. In our previous computational experiments, to avoid such a subjective trap, we took a strategy to investigate objective necessary conditions of consciousness. Our basic hypothesis was that predictive internal dynamics serves as such a condition. This is in line with theories of consciousness that treat retention (memory), protention (anticipation), and primary impression as the tripartite temporal structure of consciousness. To test our hypothesis, we analyzed publicly available sleep and awake electroencephalogram (EEG) data. Our results show that EEG signals from awake or rapid eye movement (REM) sleep states have more predictable dynamics compared to those from slow-wave sleep (SWS). Since awakeness and REM sleep are associated with conscious states and SWS with unconscious or less consciousness states, these results support our hypothesis. The results suggest an intricate relationship among prediction, consciousness, and time, with potential applications to time perception and neurorobotics.

## 1. Introduction

Consciousness is a complex and multi-faceted phenomenon defying scientific explanation. However, recent advances in neuroscience methods and increased attention to the phenomenon have led to serious scientific investigations of the subject (Edelman, 1989; Crick, 1994; Koch, 2007). Features and aspects of consciousness include first-person nature, qualitative character, phenomenal structure, subjectivity, self-perspectival organization, unity, intentionality transparency, and dynamic flow (Van Gulick, 2004).

In this paper, we will focus on the dynamic, temporal nature of consciousness (James, 1890). Time is central to brain function since most of the major functions of the brain such as memory (Shastri, 2002), prediction (Henn, 1987; Gross et al., 1999; Rao and Sejnowski, 2000a; Kozma and Freeman, 2003), and motor action (Graziano et al., 2002) unfold over time. Furthermore there are temporal mechanisms at all scales, from fast and slow synaptic dynamics (Markram et al., 1997; Bi and Poo, 1998) to recurrent long-range projections among cortical and subcortical brain regions (Felleman and Van Essen, 1991; Douglas et al., 1995), and long-term plasticity that form the basis of organismal memory (Artola and Singer, 1987). Many of these temporal properties can potentially contribute to consciousness, but in this work we will specifically investigate the relationship between predictive dynamics and conscious states. Prediction has gained increasing interest from researchers as one of the central functions of the brain (Wolpert et al., 1995, 1998; Möller, 1997; Gross et al., 1999; Kawato, 1999; Witney et al., 1999; Rao and Sejnowski, 2000b; Wolpert and Flanagan, 2001; Diedrichsen et al., 2003; Bongard et al., 2006). However, in the works above, prediction has not been directly associated with consciousness. Husserl (1966) was perhaps the first to notice the relationship between prediction and consciousness, and proposed that consciousness is based on a tripartite temporal structure that includes retention (memory), protention (anticipation or prediction), and primary impression (see chapter 6 in Dainton, 2006). Here we will focus on the predictive aspect of conscious states.

In our previous computer simulation works, we argued that predictable internal brain dynamic is a necessary condition of consciousness (Kwon and Choe, 2008; Choe et al., 2012; Chung et al., 2012). The argument was based on agency, self-awareness, and high predictability of self-authored actions. Experimental and theoretical support exists for this idea. For example, Daprati et al. (1997) reviews experiments relating agency and anticipation of own action, and Hesslow (2002) suggested that simulation of action relates to conscious thought (also see experiments on body-ownership reported by Tsakiris et al., 2007).

In this article, we tested the hypothesis that predictable internal brain dynamics are correlated with conscious states. We analyzed public electroencephalogram (EEG) data from the PhysioBank (Goldberger et al., 2000) to test our hypothesis. We took the EEG data taken during awake and sleeping states, both slow-wave sleep (SWS) and rapid eye movement (REM) sleep, and measured the predictability in their dynamics. These three states are associated with varying degree of consciousness, thus they serve as a good testing ground for our analysis. Awake state is conscious by definition, and REM state also generally considered conscious mainly due to lucid dreams during REM sleep (LaBerge et al., 1981). Dreams do occur during SWS but compared to REM sleep, memory and details lags behind significantly (Cavallero et al., 1992), thus consciousness seems to be reduced or abolished. Our results based on predicting inter-peak interval (IPI) in the EEG signals turned out to be consistent with our hypothesis. Conscious states (awake or REM sleep) showed high predictability while unconscious (or less conscious) states (SWS) low predictability, i.e., awake and REM sleep EEG data exhibited high predictability while SWS EEG data showed low predictability.

In the following, we will present our EEG data analysis method and the results, and discuss limitations of our findings and their implications on time perception and neurorobotics.

## 2. Materials and methods

### 2.1. EEG data

For our analysis, we used the EEG data from PhysioBank. PhysioBank is a free online database that has a large, growing collection of digitized physiological signals and related data for the biomedical research community (Goldberger et al., 2000). The particular data set we used is the Sleep-EDF Database which includes recordings obtained from Caucasian males and females (21–35 years old) under no medication. The recordings contain horizontal EOG, Fpz-Cz, and PzOz EEG, sampled at 100 Hz. The details of the Sleep-EDF Database is described in Kemp et al. (2000). Among these data sets, we used the Fpz-Cz EEG data of the four subjects (two males and two females) from the database. An EEG amplifier measures voltage difference between different points on the scalp. The Fpz-Cz EEG is the measure of the two electrodes that are located at the Fpz (above the nasion) and the Cz position (top of the head), respectively.

### 2.2. EEG data analysis

**Figure 1:**
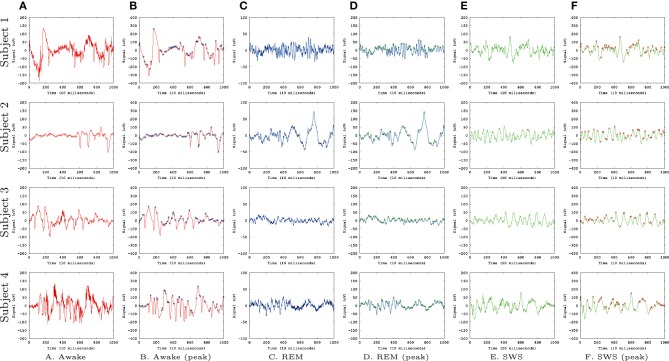
**EEG Data**. EEG data (Kemp et al., 2000) from the PhysioBank (Goldberger et al., 2000) are shown. Each row represents data from each subject (four total) and each column represents different states. **(A)** Awake, raw data. **(B)** Awake, smoothed (Gaussian filter, σ = 1), and peaks identified (circles). **(C)** REM, raw data. **(D)** REM, smoothed and peaks identified. **(E)** SWS, raw data. **(F)** SWS, smoothed and peaks identified. Each data set had 30,000 data points but here we are showing only the first 1000 for a better view of the details.

Figure 1 shows the EEG data sets we used for our analysis, from Kemp et al. (2000). We used EEG signals from four subjects with recordings during awake state **(A,B)**, REM sleep **(C,D)**, and SWS **(E,F)**. We convolved the EEG signal with a Gaussian filter with σ = 1 to smooth the signals. This was done to avoid sharp, high frequency peaks that made prediction difficult in all conditions (awake, REM, and SWS).

We used a multi-layer neural network to measure the predictability of the EEG time series data. The idea is to train a neural network to predict future data points in the EEG time series, based on data points in the past. A more predictable data set will result in lower training error. Neural networks like these have been shown to be effective non-linear predictors for time-varying signals (Principe et al., 1992). Due to the non-linear property, compared to linear predictors such as autoregressive models (Blinowska and Malinowski, 1991), neural networks are known to give generally better performance (Coyle et al., 2005). Note, however, that the particular type of algorithm used to measure predictability is not of central importance and we expect similar results with any other reasonable algorithm.

**Figure 2:**
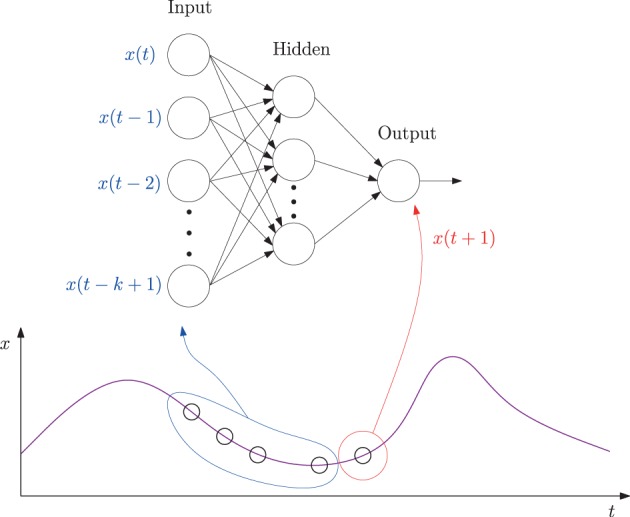
**A neural network predictor for time series data**. A multi-layer neural network consisting of *k* = 10 input units, 10 hidden units, and one output unit was trained. The input values were taken from *k* consecutive values from a time series leading up to time *t* (time step *t* − *k* + 1 to *t*), and the target output value set to the value at time step *t* + 1. The network is activated in a feed-forward manner, through the connections, and the error in the output vs. the target value back propagated to adjust the connection weights. See the text for more details.

The specific method we used was based on our earlier work reported in Kwon and Choe (2008), with one minor difference that exact error values were measured in this study instead of using the adaptive error rates. We trained a multi-layer neural network where the inputs are *k* past data points (*k* = 10 in our case) and the target output is the current data point in each EEG time series (Figure 2). Each EEG time series was traversed with a window of size 10 to construct the input set and the value immediately following the time window was used as the target value, thus forming the data set.

Using the neural network predictor described in Figure 2, given an EEG data sequence, we measured how predictable the *k* + 1-th data point is, given the past *k* data points. For each data set, a separate neural network was trained. The network was trained using the Levenberg–Marquardt algorithm, following Hagan and Menhaj (1994). In the algorithm, a damping parameter λ determines how much the algorithm will approximate Newton's method (small λ) or gradient descent (large λ). The parameter λ was initially set to 0.001 with its decrease factor set to 0.1 and increase factor to 10 (for details on λ adjustment, see Hagan and Menhaj, 1994).

Initially, we applied the above approach to predict the convolved EEG time series directly. However, we were not able to find any significant difference in predictability across the three different conditions. Based on our successful pilot analysis of single neuron recording (spike train) data, where we were able to predict the inter-spike interval (ISI), we considered detecting the EEG signal peaks and predicting the inter-peak interval (IPI), or inter-peak distance (Tyner and Knott, 1983, p. 83). Please refer to the Discussion section for more information regarding neuronal ISI prediction and why we did not include those results here.

To measure IPI, we used a simple local search (whether data point at *t* has a higher value than its immediate neighbors at *t* − 1 and *t* + 1) to detect the local peaks in the convolved EEG data (Figures 1B,D,F, marked with circles). From these peak locations, we calculated the inter-peak interval (IPI), and collected a sequence of IPI values for each EEG data set. A unique neural network was trained for each of the 12 combinations of experimental subject and conscious state (Awake, REM, SWS). To train and test each network, the IPI time series was calculated from the relevant EEG dataset, and split into training set (60%), validation set (15%), and test set (25%).

## 3. Results

**Figure 3:**
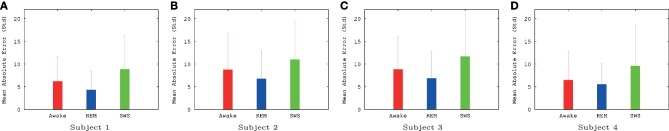
**Summary of EEG IPI prediction error results (mean and standard deviation)**. Mean and standard deviation of IPI prediction error are shown for all four subjects, for all three conditions (awake, REM, and SWS). The unit for the *y*-axis was 10 ms. For all subjects, awake and REM conditions resulted in lower IPI prediction error than SWS, showing that predictive dynamics may be more prominent during conscious states. All differences were significant (*t*-test, *p* < 10^−6^), except for REM vs. AWAKE for subject 4. See text for details. Awake state having higher IPI prediction error than REM state is somewhat unexpected, which we will discuss further in the Discussion section. **(A)** Subject 1, **(B)** subject 2, **(C)** subject 3, **(D)** subject 4.

**Figure 4:**
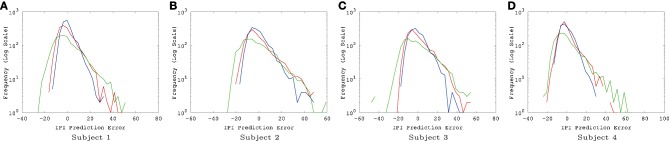
**EEG IPI prediction error distribution**. The IPI prediction error distribution is shown for all four subjects, each for all three conditions (awake [red], REM [blue], and SWS [green]). The *x*-axis is in linear scale while the *y*-axis is in log scale for a clearer view of the probability of extreme error values. The unit for the *x*-axis was 10 ms. The trends are consistent for all four subjects. REM has the highest peak near zero error, closely followed by awake state, and finally SWS which shows the lowest peak. SWS has the heaviest tail, meaning that high error values are much more common than awake state or REM. **(A)** Subject 1, **(B)** subject 2, **(C)** subject 3, **(D)** subject 4.

The IPI prediction error on novel data (not used during training or validation) are summarized in Figure 3 and the error distributions shown in Figure 4.

The results show that, for all four subjects, on average, both awake state and REM have lower IPI prediction error than SWS (Figure 3). All differences, except for REM vs. AWAKE state for subject 4, were significant (*t*-test, *p* < 10^−6^, where *n* varied [~2000] depending on how many peaks were in each data set; see below for details on statistical analysis). These results support our hypothesis regarding the predictability of internal state dynamics and conscious states (i.e., they should be correlated).

For *t*-test, the absolute error in IPI prediction was log-transformed to correct for the positive skewness of the IPI error distributions (Figure 4). The effect size (Cohen's *d*) was about medium (*d* ≥ 0.5) for all REM vs. SWS, between small and medium (0.2 ≤ *d* ≤ 0.5) for all AWAKE vs. SWS and for all REM vs. AWAKE states (except for REM vs. AWAKE for subject 4). See Table 1 for details. A medium effect size is large enough to compare means without further statistical analysis, while a small effect size requires further analysis (Cohen, 1977). The statistical power of *t*-test depends on three factors: the mean differences, the residual variance, and the sample size. Given a fixed Cohen's *d*, increasing sample size improves statistical power; since the degrees of freedom of the *t*-test are increased, the mean differences do not need to be as large to be significant (Kenny, 1987). Based on this, we can assert the main interpretation above.

**Table 1:** **Effect size (Cohen's d)**.

	**REM vs. SWS**	**Awake vs. SWS**	**REM vs. Awake**
Subject 1	0.6605	0.3173	0.3647
Subject 2	0.5104	0.2013	0.3225
Subject 3	0.5027	0.2515	0.2586
Subject 4	0.4534	0.3927	0.0368

**Figure 5:**
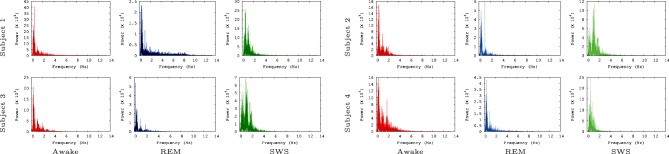
**FFT power spectrum of the raw EEG data**. FFT power spectrum of each EEG data set is shown. Most peaks are observed near 1 Hz and 2 Hz. Note that the results shown here are based on the raw EEG data, not the IPI data, and that the *y*-axis are scaled differently to fit the data.

We also ran the Fast Fourier Transform (FFT) power spectral analysis with the EEG data to rule out the possibility that of our findings simply reflect the varying power of alpha waves in the three tested conditions. Alpha waves are in the frequency range of 7.5–12.5 Hz (Berger, 1929) and are known for synchronous, and coherent sinusoidal oscillations in EEG brain signals (Nunez et al., 2001; Gerrard and Malcolm, 2007). Therefore, alpha waves are probably most predictable neural oscillations in EEG brain signals. In our FFT power spectral analysis results (Figure 5), alpha waves were not notably observed for all data, even in the awake states. This is partly because alpha waves are reduced with open eyes, drowsiness, and sleep. Note that in the EEG data we analyzed the participants were conducting normal activity at home with open eyes when the AWAKE EEG data were recorded. Therefore, it seems that there is no strong association between IPI prediction and the alpha wave spectral power in the EEG data.

**Figure 6:**
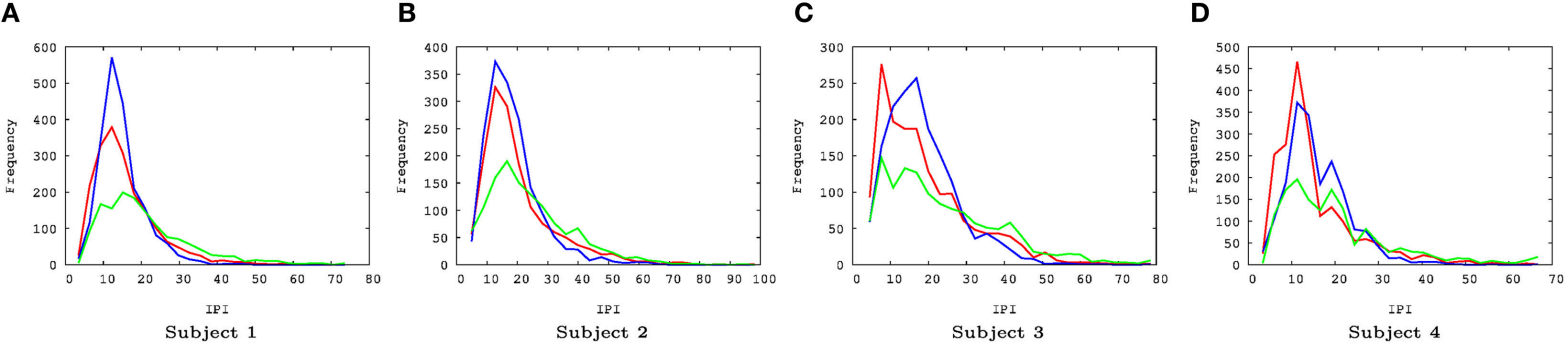
**EEG IPI distribution**. **(A–D)** The IPI distributions are shown for all four subjects, for all three conditions (awake [red], REM [blue], and SWS [green]). For all cases, the IPI distributions are positively skewed. The skewness varied from 0.83 to 2.71. The *x*-axis represents time (unit = 10 ms) and the *y*-axis frequency. **(A)** Subject 1, **(B)** subject 2, **(C)** subject 3, **(D)** subject 4.

There were a couple of interesting properties we observed in the results. First, REM data had the lowest IPI prediction error, even compared to the awake state. This was somewhat unexpected since we hypothesized predictability will be correlated with the degree of consciousness and by default we expected that the awake state is the most conscious. This is an interesting counterintuitive result. Second, all error distributions have a broader spread toward positive error, relative to negative error (i.e., the distribution is positively skewed, with skewness ranging from 0.86 to 1.69, Figure 4). Since the error is calculated as *error* = *true* − *predicted*, underestimation of the IPI seems more error-prone than overestimation. This could be due to the skewness in the IPI distribution itself (Figure 6): See the Discussion section for a detailed discussion on both phenomena.

## 4. Discussion

In this article, we analyzed publicly available EEG data from sleep and awake states to measure the predictability of the signals under conscious (awake and REM sleep) and unconscious (SWS) conditions. We found that the predictability of EEG signals correlated with the degree of consciousness. These results support our earlier hypothesis that predictable internal brain dynamics is a necessary condition of consciousness. In the following, we will discuss potential issues and interesting observations from our study, and propose potential applications of our finding to time perception and neurorobotics.

There are potential limitations of our approach as we briefly mentioned in the Materials and Methods section. We measured predictability in the inter-peak interval in the EEG signals, not directly on the raw EEG signals. Predictability measured on raw EEG signals did not show any significant differences among the three conditions: awake, REM, and SWS (pilot results, data not shown). This could be due to multiple factors, one of which is the nature of the EEG signals. For example, EEG signals are weighted mixtures of on-going electrical activity in the brain. Also, generally reduced levels of activity during SWS may result in flatter signals (slowly changing and low-amplitude, further confounded by mixing) which may be easier to extrapolate from. Based on this observation, we initially analyzed single neuron spike train data obtained during sleep and awake states by Steriade et al. (2001). Using the data, we used the same feed-forward neural network predictor to predict the inter-spike interval (ISI) under awake, REM, and SWS conditions. Our results were consistent with what we reported here, however, the data set was very small (on the order of 100 spikes per condition, compared to thousands of peaks in the EEG data) so we could not draw meaningful conclusions. However, since we found that using discrete events (spikes) instead of the continuous wave form gave promising results, we tried to recover such events in the EEG data which led us to the inter-peak interval (IPI) measure. (Note that the above is simply our motivation to use EEG IPI, and not a claim that we are extracting spike timing information from the EEG signals.) Aside from the dynamic data we discussed above (raw EEG, IPI based on EEG, or ISI), event-related potential (ERP) could have been analyzed. However, ERPs are by definition event-related, thus they are anchored to specific tasks or stimuli. Furthermore, ERPs are averages of over large number of trials. Due to these reasons, ERPs may not be suitable for studying ongoing baseline states such as awake, dreaming, or sleep, although they may be effective in detecting transition events between these on-going states (Ogilvie et al., 1991).

One rather unexpected result was that the IPI prediction error was lower for REM sleep than awake state, and significantly so [*t*-test, *p* < 10^−6^ in all cases (except for REM vs. AWAKE for subject 4)]. Does this mean that subjects are more conscious during REM sleep than when they are awake? The reason for this may again be due to the mixed nature of EEG signals, plus the natural sources of randomness in the stimulus environment during the awake state. Because the awake EEG signals are driven both by the internal brain dynamics and the external stimuli, a mixture of the two may be slightly less predictable. A possible way to isolate the internal vs. external sources would be to use blind source separation, e.g., independent components analysis (Delorme and Makeig, 2004), and correlate the isolated components with the stimulus statistics. This way, we can rule out the externally driven signal variability during awake state. Our prediction is that the predictability of these internal components would be as high as that of the REM data.

Another interesting property of the IPI prediction error distribution is its positive skewness under all conditions (Figure 4). Positive skewness means more positive error than negative error, which indicates underestimation of IPI (since *error* = *true* − *predicted*). One possible explanation for this is that the prediction mechanism may be tuned more to shorter IPIs as the EEG signals generally tend to show high-frequency bursts followed by occasional pause of low-frequency intervals. The IPI distribution itself (Figure 6) shows that, for all cases, the distributions are positively skewed, and so the number of IPI values smaller than the mean is more frequent than those with values larger than the mean. This trend can explain the positive skewness of the IPI prediction error.

Finally, we would like to discuss briefly some implications of our results on time perception and neurorobotics. Our main findings were (1) the existence of predictable dynamics and its relation to conscious states, and (2) its discrete (peak to peak event) and slow (~100 to 150 ms, compared to action potentials) nature (Figure 6). First, the very existence of such regular and predictable internal dynamics could be a foundation for time perception mechanisms, for example, as a pace maker or a internal metric against which order and duration (Wittmann and Paulus, 2008; Maniadakis et al., 2009) can be inferred. Second, the discrete and slow nature of such predictable dynamics could be well suited to behavior and cognition, by providing partitionings in perceived internal time that correspond to behavioral/cognitive time scales. A deeper understanding of this connection can lead to robust time perception and control mechanisms for neurorobotics.

### Conflict of interest statement

The authors declare that the research was conducted in the absence of any commercial or financial relationships that could be construed as a potential conflict of interest.

## References

[B1] Artola A. Singer W. (1987). Long-term potentiation and NMDA receptors in rat visual cortex. Nature 330, 649–652 10.1038/330649a0 2446147

[B2] Berger H. (1929). Über das elektrenkephalogramm des menschen. Eur. Arch. Psychiatry Clin. Neurosci. 87, 527–570

[B3] Bi G.-Q. Poo M.-M. (1998). Activity-induced synaptic modifications in hippocampal culture: dependence on spike timing, synaptic strength and cell type. J. Neurosci. 18, 10464–10472 9852584 10.1523/JNEUROSCI.18-24-10464.1998 PMC6793365

[B4] Blinowska K. Malinowski M. (1991). Non-linear and linear forecasting of the eeg time series. Biol. Cybern. 66, 159–165 10.1007/BF00243291 1768720

[B5] Bongard J. Zykov V. Lipson H. (2006). Resilient machines through continuous self-modeling. Science 314, 1118–1121 10.1126/science.1133687 17110570

[B6] Cavallero C. Cicogna P. Natale V. Occhionero M. Zito A. (1992). Slow wave sleep dreaming. Sleep 15, 562–566 1475572 10.1093/sleep/15.6.562

[B7] Choe Y. Kwon J. Chung J. R. (2012). Time, consciousness, and mind uploading. Int. J. Mach. Conscious. 4, 257–274 10.1142/S179384301240015X

[B8] Chung J. R. Kwon J. Mann T. A. Choe Y. (2012). Evolution of time in neural networks: from the present to the past, and forward to the future, in The Relevance of the Time Domain to Neural Network Models. Springer series in cognitive and neural systems, Vol. 3, eds Rao A. R. Cecchi G. A. (New York, NY: Springer), 99–116

[B9] Cohen J. (1977). Statistical Power Analysis for the Behavioral Sciences. Hillsdale, NJ: Lawrence Erlbaum Associates, Inc

[B10] Coyle D. Prasad G. McGinnity T. M. (2005). A time-series prediction approach for feature extraction in a brain-computer interface. IEEE Trans. Neural Syst. Rehabil. Eng. 13, 461–467 10.1109/TNSRE.2005.857690 16425827

[B11] Crick F. (1994). The Astonishing Hypothesis: The Scientific Search for the Soul. New York, NY: Charles Scribner's Sons

[B12] Dainton B. (2006). Stream of Consciousness: Unity and Continuity in Conscious Experience. Abingdon: Routledge (imprint of Taylor & Francis)

[B13] Daprati E. Franck N. Georgieff N. Proust J. Pacherie E. Dalery J. (1997). Looking for the agent: an investigation into consciousness of action and self-consciousness in schizophrenic patients. Cognition 65, 71–86 10.1016/S0010-0277(97)00039-5 9455171

[B14] Delorme A. Makeig S. (2004). EEGLAB: an open source toolbox for analysis of single-trial EEG dynamics including independent component analysis. J. Neurosci. Methods 134, 9–21 10.1016/j.jneumeth.2003.10.009 15102499

[B15] Diedrichsen J. Verstynen T. Hon A. Lehman S. L. Ivry R. B. (2003). Anticipatory adjustments in the unloading task: is an efference copy necessary for learning? Exp. Brain Res. 148, 272–276 10.1007/s00221-002-1318-z 12520418

[B16] Douglas R. J. Koch C. Mahowald M. Martin K. A. C. Suarez H. H. (1995). Recurrent excitation in neocortical circuits. Science 269, 981–985 10.1126/science.7638624 7638624

[B17] Edelman G. M. (1989). The Remembered Present: A Biological Theory of Consciousness. New York, NY: Basic Books

[B18] Felleman D. J. Van Essen D. C. (1991). Distributed hierarchical processing in primate cerebral cortex. Cereb. Cortex 1, 1–47 10.1093/cercor/1.1.1 1822724

[B19] Gerrard P. Malcolm R. (2007). Mechanisms of modafinil: a review of current research. Neuropsychiat. Dis. Treat. 3, 349–364 19300566 PMC2654794

[B20] Goldberger A. L. Amaral L. A. Glass L. Hausdorff J. M. Ivanov P. C. Mark R. G. (2000). Physiobank, physiotoolkit, and physionet components of a new research resource for complex physiologic signals. Circulation 101, e215–e220 10.1161/01.CIR.101.23.e215 10851218

[B21] Graziano M. S. A. Taylor C. S. R. Moore T. (2002). Complex movements evolved by microstimulation of precentral cortex. Neuron 34, 841–851 10.1016/S0896-6273(02)00698-0 12062029

[B22] Gross H.-M. Heinze A. Seiler T. Stephan V. (1999). Generative character of perception: a neural architecture for sensorimotor anticipation. Neural Netw. 12, 1101–1129 10.1016/S0893-6080(99)00047-7 12662648

[B23] Hagan M. T. Menhaj M. B. (1994). Training feedforward networks with the Marquadt algorithm. IEEE Trans. Neural Netw. 5, 989–993 10.1109/72.329697 18267874

[B24] Henn V. (1987). History of cybernetics, in The Oxford Companion to the Mind, ed. Gregory R. L. (Oxford: Oxford University Press), 174–177

[B25] Hesslow G. (2002). Conscious thought as simulation of behaviour and perception. Trends Cogn. Sci. 6, 242–247 10.1016/S1364-6613(02)01913-7 12039605

[B26] Husserl E. (1966). The Phenomenology of Internal Time-Consciousness. Bloomington, IN: Indiana University Press

[B27] James W. (1890). The Principles of Psychology. New York, NY: Henry Holt 10.1037/11059-000

[B28] Kawato M. (1999). Internal models for motor control and trajectory planning. Curr. Opin. Neurobiol. 9, 718–727 10.1016/S0959-4388(99)00028-8 10607637

[B29] Kemp B. Zwinderman A. H. Tuk B. Kamphuisen H. A. Oberye J. J. (2000). Analysis of a sleep-dependent neuronal feedback loop: the slow-wave microcontinuity of the EEG. IEEE Trans. Biomed. Eng. 47, 1185–1194 10.1109/10.867928 11008419

[B30] Kenny D. A. (1987). Statistics for the Social and Behavioral Sciences. Boston, MA: Little, Brown

[B31] Koch C. (2007). The Quest for Consciousness: A Neurobiological Approach. Greenwood Village, CO: Roberts & Company Publishers

[B32] Kozma R. Freeman W. J. (2003). Basic principles of the KIV model and its application to the navigation problem. J. Integr. Neurosci. 2, 125–145 10.1142/S0219635203000159 15011280

[B33] Kwon J. Choe Y. (2008). Internal state predictability as an evolutionary precursor of self-awareness and agency, in Proceedings of the Seventh International Conference on Development and Learning (IEEE) (Monterey, CA), 109–114

[B34] LaBerge S. P. Nagel L. E. Dement W. C. Zarcone V. P. Jr. (1981). Lucid dreaming verified by volitional communication during rem sleep. Percept. Motor Skills 52, 727–732 10.2466/pms.1981.52.3.727 24171230

[B35] Maniadakis M. Trahanias P. Tani J. (2009). Explorations on artificial time perception. Neural Netw. 22, 509–517 10.1016/j.neunet.2009.06.045 19619980

[B36] Markram H. Lübke J. Frotscher M. Sakmann B. (1997). Regulation of synaptic efficacy by coincidence of postsynaptic APs and EPSPs. Science 275, 213–215 10.1126/science.275.5297.213 8985014

[B37] Möller R. (1997). Perception through anticipation: an approach to behavior-based perception, in Proceedings of New Trends in Cognitive Science, 184–190

[B38] Nunez P. L. Wingeier B. M. Silberstein R. B. (2001). Spatial-temporal structures of human alpha rhythms: theory, microcurrent sources, multiscale measurements, and global binding of local networks. Hum. Brain Mapp. 13, 125–164 10.1002/hbm.1030 11376500 PMC6872048

[B39] Ogilvie R. D. Simons I. A. Kuderian R. H. MacDonald T. Rustenburg J. (1991). Behavioral, event-related potential, and EEG/FFT changes at sleep onset. Psychophysiology 28, 54–64 10.1111/j.1469-8986.1991.tb03386.x 1886964

[B40] Principe J. C. Rathie A. Kuo J.-M. (1992). Prediction of chaotic time series with neural networks and the issue of dynamic modeling. Int. J. Bifurcat. Chaos 2, 989–996 10.1142/S0218127492000598

[B41] Rao R. P. Sejnowski T. J. (2000a). Predictive sequence learning in recurrent neocortical circuits, in Advances in Neural Information Processing Systems, Vol. 12, eds Leen T. K. Dietterich T. G. Tresp V. (Cambridge, MA: MIT Press), 164–170

[B42] Rao R. P. N. Sejnowski T. J. (2000b). Predictive sequence learning in recurrent neocortical circuits, in Advances in Neural Information Processing Systems, Vol. 13, eds Solla S. A. Leen R. K. Muller K.-R. (Cambridge, MA: MIT Press), 164–170

[B43] Shastri L. (2002). Episodic memory and cortico-hippocampal interactions. Trends Cogn. Sci. 6, 162–168 10.1016/S1364-6613(02)01868-5 11912039

[B44] Steriade M. Timofeev I. Grenier F. (2001). Natural waking and sleep states: a view from inside neocortical neurons. J. Neurophysiol. 85, 1969–1985 11353014 10.1152/jn.2001.85.5.1969

[B45] Tsakiris M. Hesse M. D. Boy C. Haggard P. Fink G. R. (2007). Neural signatures of body ownership: a sensory network for bodily self-consciousness. Cereb. Cortex 17, 2235–2244 10.1093/cercor/bhl131 17138596

[B46] Tyner F. S. Knott J. R. (1983). Fundamentals of EEG Technology. Basic concepts and methods, Vol. 1 Philadelphia, PA: Lippincott Williams & Wilkins

[B47] Van Gulick R. (2004). Consciousness, in Stanford Encyclopedia of Philosophy, ed Zalta E. N. (Stanford, CA: Stanford University). Available online at: http://plato.stanford.edu/entries/consciousness/

[B48] Witney A. Goodbody S. J. Wolpert D. M. (1999). Predictive motor learning of temporal delays. J. Neurophysiol. 82, 2039–2048 10561385 10.1152/jn.1999.82.5.2039

[B49] Wittmann M. Paulus M. P. (2008). Decision making, impulsivity and time perception. Trends Cogn. Sci. 12, 7–12 10.1016/j.tics.2007.10.004 18042423

[B50] Wolpert D. M. Flanagan J. R. (2001). Motor prediction. Curr. Biol. 11, R729–R732 10.1016/S0960-9822(01)00432-8 11566114

[B51] Wolpert D. M. Ghahramani Z. Jordan M. I. (1995). An internal model for sensorimotor integration. Science 269, 1880–1882 10.1126/science.7569931 7569931

[B52] Wolpert D. M. Miall R. C. Kawato M. (1998). Internal models in the cerebellum. Trends Cogn. Sci. 2, 338–347 10.1016/S1364-6613(98)01221-2 21227230

